# NFAT5 mediates hypertonic stress-induced atherosclerosis via activating NLRP3 inflammasome in endothelium

**DOI:** 10.1186/s12964-019-0406-7

**Published:** 2019-08-20

**Authors:** Pingping Ma, Shenfang Zha, Xinkun Shen, Yulan Zhao, Li Li, Li Yang, Mingxing Lei, Wanqian Liu

**Affiliations:** 1 Key Laboratory of Biorheological Science and Technology, Ministry of Education, Bioengineering College, Chongqing University, Chongqing, 400044 China; 2 Integrative Stem Cell Center, China Medical University Hospital, China Medical University, Taichung, 40402 Taiwan; 3 Institute of New Drug Development, College of Biopharmaceutical and Food Sciences, China Medical University, Taichung, 40402 Taiwan

**Keywords:** Atherosclerosis, NLRP3 inflammasome, NFAT5, Hypertonic stress, Endothelium

## Abstract

**Background:**

How high-salt intake leads to the occurrence of many cardiovascular diseases such as atherosclerosis is a fundamental question in pathology. Here we postulated that high-salt-induced NFAT5 controls the inflammasome activation by directly regulating NLRP3, which mediates the expression of inflammatory- and adhesion-related genes in vascular endothelium, resulting in the formation of atherosclerosis.

**Methods:**

Atherosclerosis-prone apolipoprotein E-deficient (ApoE−/−) mice which accumulate cholesterol ester-enriched particles in the blood due to poor lipoprotein clearance capacity were used as the atherosclerosis model in vivo. Cultured endothelial cells (ECs) and monocytes under high-salt condition were used to explore the atheroprone role of the activation of NFAT5-NLRP3 inflammasome in vascular endothelium in vitro. Bioinformatic analysis and chromatin immunoprecipitation assay were used to identify the DNA binding sites of NFAT5 on promoters of NLRP3 and IL-1β.

**Results:**

We first observe that high-salt intake promotes atherosclerosis formation in the aortas of ApoE^−/−^ mice, through inducing the expression of NFAT5, NLRP3, and IL-1β in endothelium. Overexpression of NFAT5 activates NLRP3-inflammasome and increases the secretion of IL-1β in ECs partly via ROS. Chromatin immunoprecipitation assay demonstrates that NFAT5 directly binds to the promoter regions of NLRP3 and IL-1β in endothelial cells subjected to the high-salt environment.

**Conclusions:**

Our study identifies NFAT5 as a new and essential transcription factor that is required for the early activation of NLRP3-inflammasome-mediated endothelium innate immunity, contributing to the formation of atherosclerosis under hypertonic stress induction.

**Electronic supplementary material:**

The online version of this article (10.1186/s12964-019-0406-7) contains supplementary material, which is available to authorized users.

## Background

Atherosclerosis (AS), a leading cause of cardiovascular morbidity and mortality, is initiated and promoted by inflammation in vascular endothelium [1]. Epidemiologic studies have reported that high-salt intake, diabetes or hyperosmotic therapy, contributes to the high incidence of AS [2–4]. During AS, endothelial cells (ECs) sense cardiovascular risk factors, initiate innate immune responses, release proinflammatory cytokines and chemokines, and recruit inflammatory cells transendothelial migration into the arterial wall, resulting in the subsequent inflammation augmentation, foam cell formation, and other pathological changes [5, 6]. A common consequence caused by these risk factors is the elevated concentration of plasma sodium, which has been found to induce chronic inflammation, increase endothelial adhesive property, and accelerate macrophage infiltration and atherosclerotic plaque formation [7, 8]. High-salt reduces the release of nitric oxide (NO), increases the expression of inflammatory adhesion molecules, and potentiates the secretion of coagulation mediators in ECs [2, 9]. However, little is known about the molecular mechanism by which high-salt induces AS through endothelial activation and inflammation.

Nuclear factor of activated T cells 5 (NFAT5) has been identified as a transcription factor which orchestrates cellular defense against osmotic stress in kidney [10]. NFAT5 can be induced in tissues by various stimuli, such as high-salt, hypoxia, and mechanical stress [11–14]. NFAT5 activates the transcription of its target genes important for embryogenesis, tumor metastasis [13], and arterial stiffening [11]. Increased NFAT5 activity promotes macrophages survival and infiltration [15, 16], as well as angiogenesis [17]. Whereas NFAT5 deficiency leads to catabatic chronic inflammation and macrophage infiltration. Growing evidence has shown that NFAT5 regulates the expression of proinflammatory cytokine interleukin -1β (IL-1β) family proteins in the inflammatory disease [15, 18], and mediates the innate immune responses [19].

NLR family pyrin domain containing 3 (NLRP3) inflammasome is a crucial effector involved in innate immunity, and its activation in ECs contributes to a high risk of AS [20, 21]. Inactivation of NLRP3 inflammasome in macrophages by bone marrow-derived transplantation inhibits macrophages migration and resists early AS [22]. However, activation of the NLRP3 inflammasome by high-salt in macrophages, foam cells or other cells does not fully explain the role of EC dysfunction and inflammatory response in the formation of AS. Therefore, the impact of high-salt on endothelial NLRP3 inflammasome activation and innate immunity remains elusive.

Here, we investigate the mechanism that high-salt induces NFAT5 which regulates NLRP3 to control inflammasome activation and innate immunity in ECs. We first examined the impact of high-salt intake on atherosclerotic lesion formation, as well as endothelial inflammasome activation and inflammation in mice. Next, we determined the effects of high-salt on the expression of NLRP3, NFAT5, and IL-1β in ECs under high-salt conditions. We further explored the molecular mechanism by which NFAT5 activates NLRP3 inflammasome and increases the secretion of mature IL-1β in ECs partly via reactive oxygen species (ROS). Collectively, these findings demonstrated that NFAT5 is a master regulator of NLRP3 inflammasome activation and inflammation in ECs. Our study identified a novel and promising target for treating AS.

## Methods

### Cell culture and animal

Primary human umbilical vein endothelial cells (HUVECs) were cultured in M199 Medium (HyClone, South Logan, UT) supplemented with 3.2 ng/ml β-ECGF (Sigma, Billerica, MA), 0.108 mg/ml heparin sodium (Solarbio, Tongzhou, BJ), 2.5 ng/ml thymidine (Solarbio, Beijing), and 10% fetal bovine serum (FBS, Biological Industries, Kibbutz Beit Haemek, Israel). The osmolality of M199 medium (control) was about 270 mosmol/kg. To elevate the osmolality of M199 medium, NaCl (Sigma, Billerica, MA) was added into the control medium, with a final osmolality at 290, 310, 330, and 350 mosmol/kg. For all experiments except the long-term proliferation experiments, the logarithmically growing HUVECs between passages 2 and 6 were used. Human THP-1 monocytes were purchased from YiYuan (Guangzhou, China), and grown in RPMI 1640 medium supplemented with 10% FBS (Gibco, Grand Island, NY). Cells were maintained at 37 °C with 5% CO_2_ in a humidified atmosphere.

Eight-week male apolipoprotein E-deficient (ApoE^−/−^) mice weighed at 20~25 g were purchased from Research Institute of Surgery at Daping Hospital, the Third Military Medical University. The mice were fed with a normal salt diet (0.8% NaCl) for 2 weeks, and then divided into two groups. Group 1 was kept on with a normal salt diet (0.8% NaCl), whereas group 2 was fed with a high-salt diet (8% NaCl). All mice had free access to water.

### Oil red O staining for analysis of atherosclerotic lesions

Mice fed with normal-salt or high-salt diet for 12 weeks were euthanized. The aortas were isolated and fixed in 4% (v/v) paraformaldehyde solution for 12 h. The entire aorta was dissected longitudinally from the aortic arch (AA) to the iliac bifurcation, and stained with Oil Red O (Solarbio, Beijing) for 20 min. Images were captured under a dissecting microscope (Guangmi, China) by a digital camera (Canon, Japan). The plaques were analyzed using the Adobe PhotoShop CS6 software. The AS degree was represented as the percentage of plaque area versus the whole vascular surface area.

### siRNA transfection

HUVECs were seeded in 6-well culture plates. At a confluency of 60–70%, cells were transfected with 50 nM control or NFAT5/NLRP3 siRNA, respectively, by using Lipofectamine 2000 reagent (Invitrogen, Carlsbad, CA) in Opti-MEM serum-free media for 6 h (Invitrogen, Carlsbad, CA). Then the Opti-MEM medium was replaced by M199 complete medium with high-salt. Total RNA or total protein was extracted for the real-time qPCR or western blot analysis, respectively.

### Adenovirus infection

Recombinant adenovirus that containing the full cDNA sequence of NFAT5 (Ad-NFAT5), were purchased from Cyagen (Guangzhou, China). For adenovirus infection, HUVECs were treated with Ad-NFAT5 for 48 h before RNA or protein extraction. Adenovirus (Ad)-null infected HUVECs were used as the control group.

### RT-qPCR

HUVECs were cultured with high-salt medium for 2 days. Total RNA was extracted using the RNA simple Total RNA Kit (Tiangen, Beijing). The total RNA (1 μg) was reverse-transcribed into cDNA by using RevertAid RT Reverse Transcription Kit (Thermo Scientific, Waltham, MA). Then quantitative real-time PCR was performed on a Bio-Rad CFX96 real-time PCR system (Bio-Rad, Hercules, CA) using SsoAdvanced universal SYBR Green Supermix (Bio-Rad, Hercules, CA). The primer sequences for RT-qPCR were listed in Additional file 1: Table S1.

Aortas were collected from ApoE−/− mice fed with normal-salt or high-salt diet for 4 weeks. The tissues were minced in liquid nitrogen, and removed into tubes containing RNA lysate buffer. Total RNA was extracted using the RNA simple Total RNA Kit (Tiangen, Beijing). Expression of NLRP3, NFAT5, IL-1β, ICAM-1, VCAM-1, MCP-1, and E-selectin mRNA was examined by RT-qPCR. The primer sequences for RT-qPCR were also listed in Additional file 1: Table S1.

### Western blot

Cell lysates were subjected to electrophoretic separation by 10% SDS-PAGE gel and transferred to a PVDF membrane. Antibodies against NLRP3 (Boster, Wuhan), NFAT5 (Abcam, Cambridge, MA), IL-1β (Cell Signaling, Danvers, MA), Caspase-1 (Cell Signaling, Danvers, MA), or β-actin (Cell Signaling, Danvers, MA) diluted in the skimmed milk powder at 1:1000 were added onto the PVDF membranes and incubated at 4 °C overnight. After washing, the membranes were incubated with secondary antibodies conjugated with horseradish peroxidase for one hour. The bands were developed by using ECL substrate solution (Thermo Scientific, Waltham, MA), and analyzed with Quantity One software version 4.6.

### Chromatin immunoprecipitation (ChIP) assay

ChIP assays were performed using the Enzymatic Chromatin IP Kit (Cell Signaling, Danvers, MA) according to the manufacturer’s instructions. Three target sequences near the transcription start site of NLRP3 and IL-1β gene were designed by primer designing tools. One of these sequences contained the NFAT5 binding sequence, whereas the other two were used as negative controls.

### Immunofluorescence staining, *en face* immunostaining, and immunohistochemistry staining

HUVECs were fixed in 4% (v/v) paraformaldehyde (Solarbio, Beijing) solution at room temperature for 15 min, permeabilized with 0.2% (v/v) Triton X-100 (Solarbio, Tongzhou, BJ) for 15 min, washed with PBS three times, and blocked with 2% (w/v) BSA (Solarbio, Beijing) at 37 °C for 30 min. Then, cells were incubated with primary antibodies against NLRP3 (Boster, Wuhan) at 4 °C overnight, followed by an incubation with goat anti-rabbit Alexa Fluor 488 secondary antibodies (Beyotime, Jiangsu) at 37 °C for 30 min. Nuclei were stained with DAPI (Cell Signaling, Danvers, MA). Images were obtained by using a fluorescent microscope (Leica, Germany).

En face immunofluorescent staining was used to examine the protein expression in ECs of the aortas. The adventitial fat was removed from the arteries, which were then dissected longitudinally. Then immunostaining was performed as described above.

For immunohistochemistry staining, mice kidney were fixed in 4% (v/v) paraformaldehyde solution at 4 °C overnight, and embedded in paraffin. Sections were cut at 5 um and incubated with primary antibodies against NLRP3 (Boster, Wuhan) or ECs marker CD31 (Cell Signaling, Danvers, MA), followed by an incubation with HRP-labeled secondary antibody. The staining was developed by using a DAB substrate kit (Cell Signaling, Danvers, MA). Then, the slides were counterstained with hematoxylin (Cell Signaling, Danvers, MA). Images were taken under a microscope (Leica, Germany).

### Statistics

Shapiro-wilk test (SPSS 18.0 software) was used to evaluate the normality of experimental data before analyses (*p* > 0.05 was determined as normality). All data were confirmed to be normality and presented as mean ± SEM. Student′s t-test and ANOVA using the OriginPro (version 7.5) program were applied to the statistical analyses (*n* ≥ 3). *p* < 0.05 was considered statistically significant.

## Results

### High-salt intake predisposes atherosclerosis in aortas of ApoE^−/−^ mice

**Fig. 1:**
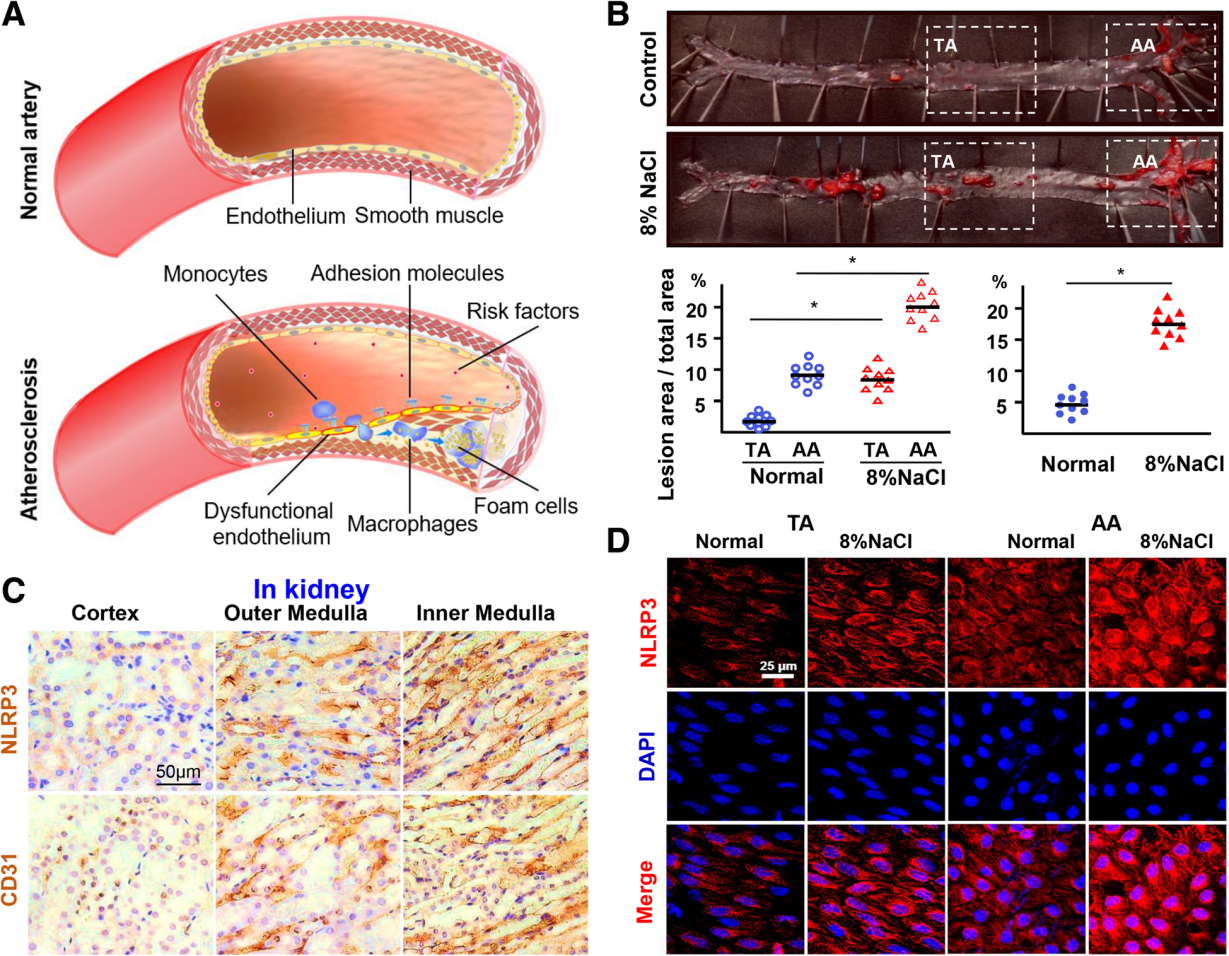
High-salt intake predisposes atherosclerosis and upregulates NLRP3 expression. **a** Schematic shows the process of atherosclerosis formation. **b** Oil Red O staining of aortas and quantification of percentage lesion areas in the thoracic aorta (TA) and aortic arch (AA) of ApoE^−/−^ mice (*n* = 10) fed with a normal or high-salt diet for 12 weeks. **c** Immunohistochemistry staining for NLRP3 and CD31 in the kidney at 4 weeks. Nuclei, hematoxylin staining. **d** *En face* immunofluorescent staining of NLRP3 of ECs in TA and AA of ApoE−/− mice fed with a normal or high-salt diet for 4 weeks. See Additional file 1: Figure S1D for NLRP3 mRNA qPCR. All data were presented as mean ± SEM, *N* ≥ 9. *p < 0.05

ApoE^−/−^ mice display a poor capability to clean the lipoprotein, resulting in an accumulation of cholesterol ester-enriched particles in the blood. Thus, ApoE^−/−^ mouse is an excellent surrogate model to study atherosclerosis. To investigate whether high-salt intake contributes to AS (Fig. 1a), we first observed atherosclerotic lesion development in ApoE^−/−^ mice that were fed with high-salt diet or normal chow for 12 weeks. Compared with the normal chow group, there was an obvious increase in body weight in the high-salt intake group (Additional file 1: Figure S1A). The serum levels of total Na^+^ and cholesterol in the high-salt intake group were significantly higher than that of the normal group (Additional file 1: Table S2). High-salt intake significantly increased atherosclerotic lesions in ApoE^−/−^ mice (Fig. 1b), particularly in the atheroprone regions of the aortic arch (AA) (Fig. 1b). H&E staining of cross-sections of the AA also showed that high-salt intake induced plaque formation in ApoE^−/−^ mice (Additional file 1: Figure S1B). These results demonstrated that high-salt intake predisposes AS.

To examine the role of high-salt intake in inducing AS, we checked macrophage infiltration on the atherosclerotic lesions by staining the macrophage marker CD-11b. Immunohistochemistry staining showed that the number of macrophages was increased in the aortic plaques of ApoE^−/−^ mice with high-salt intake, compared to the normal chow group (Additional file 1: Figure S1C). This suggests that high-salt intake promotes macrophage infiltration during AS.

### High-salt elevates NLRP3 expression in ECs

NLRP3 inflammasome not only contributes to macrophage infiltration, but also acts as a sensor of osmotic pressure [23]. We examined NLRP3 expression in ECs of different parts of kidney including renal cortex, outer and inner renal medullae, in which the osmotic pressure is gradually increased, respectively, under physiological conditions. Immunohistochemistry staining showed that the expression of NLRP3 was higher in the inner and outer renal medullae, which have a higher osmotic pressure than that in the renal cortex which has a lower osmotic pressure (Fig. 1c). This indicates that NLRP3 expression correlates with the osmotic pressure in ECs. We then investigated whether high-salt intake induces early NLRP3 inflammasome activation in the endothelium of the arterial tree in vivo. Real-time PCR result showed that the expression of NLRP3 mRNA was significantly increased in the arterial wall of the ApoE^−/−^ mice with high-salt intake than that of the normal group (Additional file 1: Figure S1D). *En face* immunofluorescent staining showed that endothelial NLRP3 expression was increased in the atheroprone regions of AA in high-salt intake group after 4 weeks than that of the normal group (Fig. 1d).

### High-salt enhances endothelial inflammation and IL-1β secretion in ECs

**Fig. 2:**
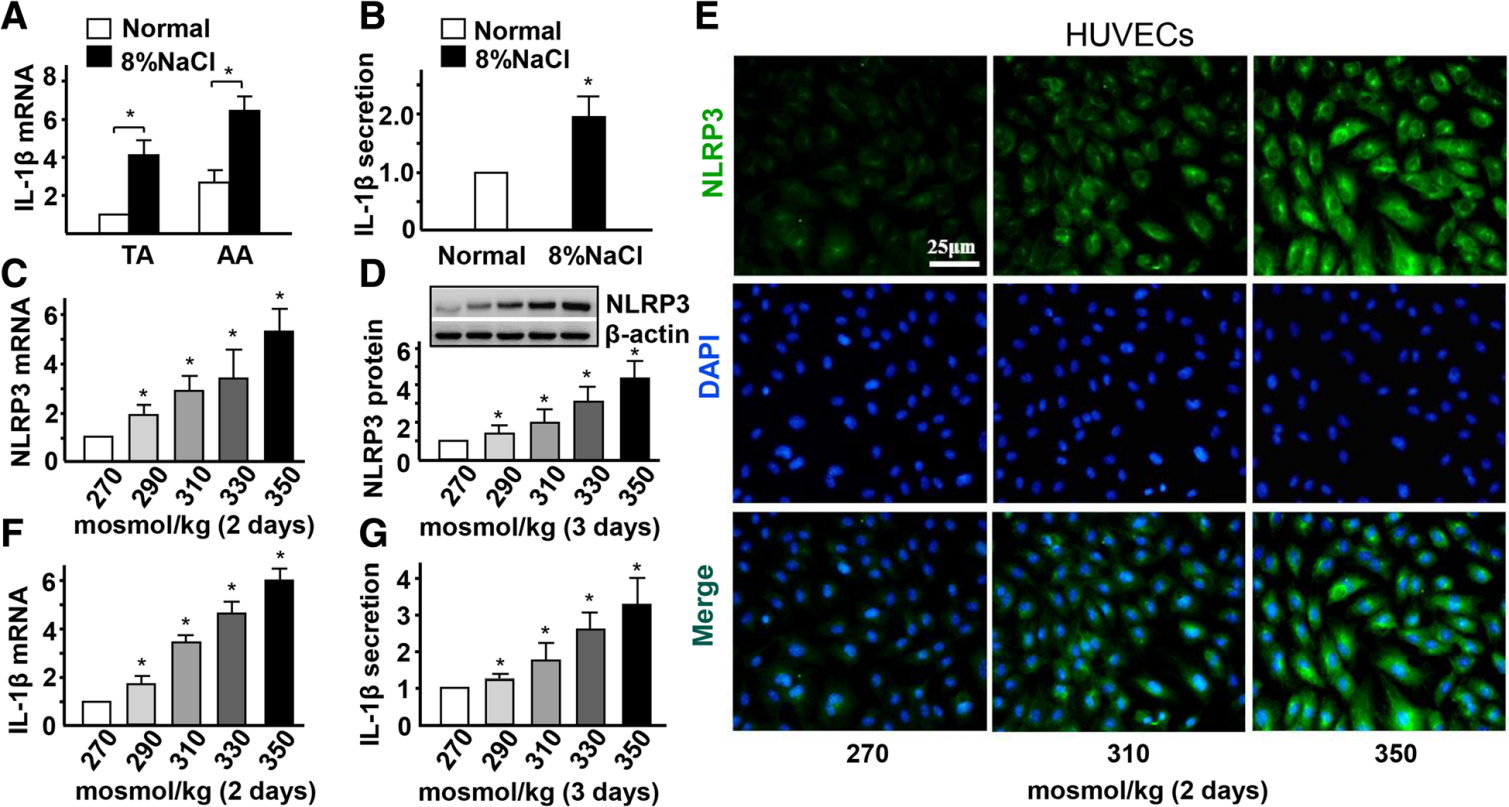
High-salt enhances NLRP3 inflammasome activation in ECs. **a** Quantification of mRNA levels of IL-1β in TA and AA of ApoE^−/−^ mice fed with a normal or high-salt diet for 4 weeks. **b** Quantification of protein levels of IL-1β in the serum of ApoE^−/−^ mice fed with a normal or high-salt diet for 4 weeks. **c, d** Quantification of mRNA levels and protein levels of NLRP3 by HUVECs in hypertonic medium (290, 310, 330 and 350 mosmol/kg) with isomolar 270 mosmol/kg as the control, evaluated by RT-qPCR and Western blotting with β-actin as the internal control. **e** Immunofluorescent staining of NLRP3 in HUVECs with iso- and hyper-osmotic media. **f, g** mRNA and protein expression of IL-1β in HUVECs exposed to iso- and hyper-osmotic media, evaluated by RT-qPCR and ELISA. All data were presented as mean ± SEM, *N* ≥ 3. *p < 0.05

To examine the early activation of NLRP3 inflammasome in endothelia of mice with high-salt intake, we checked the expression of IL-1β. Real-time PCR and ELISA assay results showed that the mRNA and protein levels of IL-1β, were significantly increased in AA and in the serum of the high-salt intake group after 4 weeks, respectively, compared to the control group (Fig. 2a-b). To test whether high-salt induces endothelial NLRP3 expression and IL-1β secretion in vitro, HUVECs were cultured in media with different osmolarities, ranging from 270 mosmol/kg (lower end of the normal physiological range) to 290, 310, 330, and 350 mosmol/kg (hypernatremia). HUVECs showed logarithmic growth for 2 weeks and maintained a normal cell phenotype when cultured with 350 mosmol/kg media (Additional file 1: Figure S2A-B). However, the expression of NLRP3 mRNA and protein was significantly increased in HUVECs cultured with high-salt medium in a dose-dependent manner (Fig. 2c-d). Immunofluorescent staining also showed that high-salt induces the expression of NLRP3 in HUVECs (Fig. 2e). In addition, real-time PCR and ELISA assay results showed that high-salt induces the expression of IL-1β mRNA and protein, respectively (Fig. 2f-g).

**Fig. 3:**
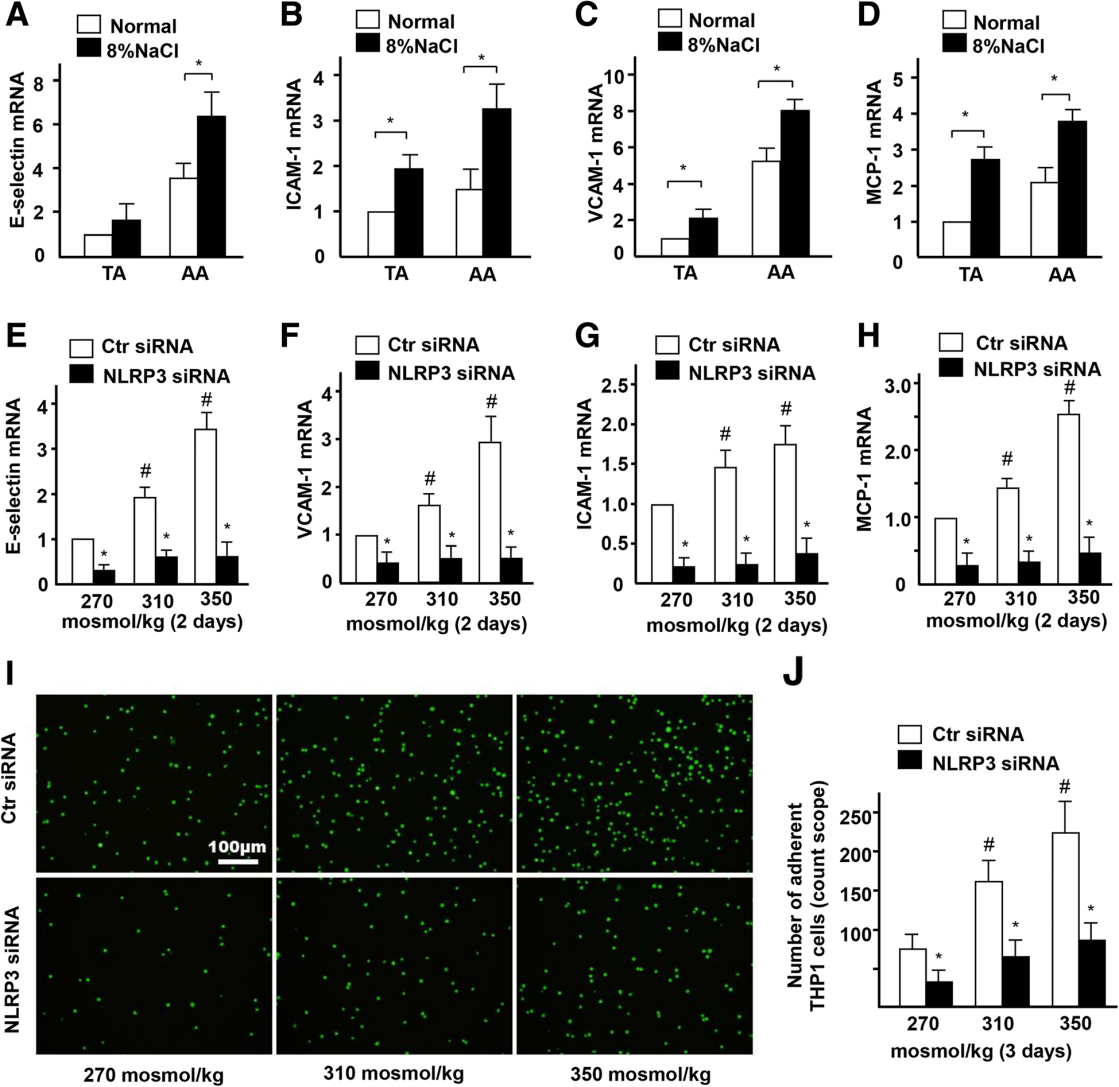
High-salt elevates endothelial inflammation via NLRP3. **a-d** Quantification of mRNA levels of E-selectin, ICAM-1, VCAM-1 and MCP-1 in TA and AA of ApoE−/− mice fed with a normal or high-salt diet. **e-h** Quantification of mRNA levels of E-selectin, VCAM-1, ICAM-1, and MCP-1 in ECs treated with high-salt and transfected with Control siRNA or NLRP3 siRNA. **i, j** The adhesion of Calcein-labeled THP-1 monocytes in ECs treated with high-salt and transfected with Control siRNA or NLRP3 siRNA. All data were presented as mean ± SEM, *N* ≥ 3. *p < 0.05

To confirm the activation of NLRP3 inflammasome and inflammation in endothelia of mice with high-salt intake for 4 weeks, we checked the expression of IL-1β downstream target genes. Real-time PCR results showed that the expression of E-selectin, VCAM-1, ICAM-1, and MCP-1 was significantly increased in the thoracic aorta (TA) and AA of the high-salt intake group, compared to the control group (Fig. 3a-d). On the contrary, real-time PCR results showed that the mRNA expression of high-salt-induced E-selectin, VCAM-1, ICAM-1, and MCP-1 was significantly decreased in HUVECs that were treated with NLPR3 siRNA (Fig. 3e-h). Fluorescence labeling and statistical analysis showed that the number of adherent monocytes increased by high-salt in ECs, was also significantly decreased when NLPR3 was suppressed by siRNA (Fig. 3i-j). Together, these results suggest that high-salt intake activates NLRP3 inflammasome-mediated ECs inflammatory response in AA.

### High-salt intake induces NFAT5 nuclear translocation in ECs

**Fig. 4:**
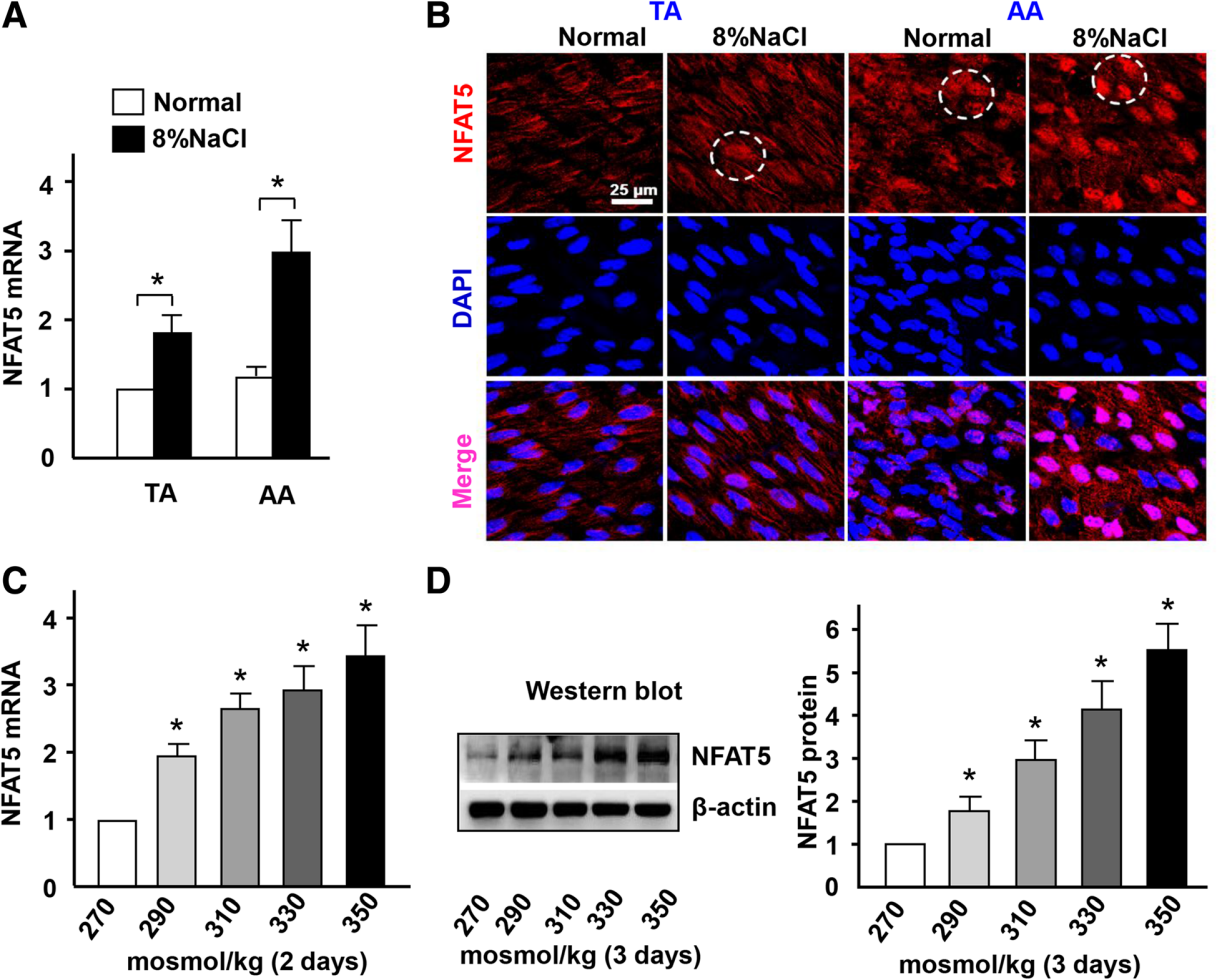
High-salt intake enhances NFAT5 expression and nuclear translocation in ECs. **a** Quantification of mRNA levels of NFAT5 in TA and AA of ApoE−/− mice fed with a normal or high-salt diet. **b** *En face* immunofluorescent staining of NFAT5 in ECs of TA and AA of ApoE−/− mice fed with a normal or high-salt diet. Circles mark the cells with nuclear NFAT5 expression. **c, d** mRNA and protein levels of NFAT5 in HUVECs exposed to iso- and hyper-osmotic media, evaluated by RT-qPCR and western blotting with β-actin as the internal control. All data were presented as mean ± SEM, *N* ≥ 3. *p < 0.05

NFAT5 has been identified as a transcription factor that can be activated by osmolality [11]. We examined the effect of high-salt intake on the expression of NFAT5 in the artery. Real-time PCR result revealed that the expression of NFAT5 mRNA was significantly increased in the arterial wall of the ApoE^−/−^ mice with high-salt intake at 4 weeks than that of the normal group (Fig. 4a). *En face* immunofluorescent staining showed that NFAT5 expression was increased in the atheroprone regions of AA in the high-salt intake group than that of the normal group (Fig. 4b). In addition, NFAT5 expression was translocated into the nucleus of ECs in high-salt intake group than that of the normal group, in which NFAT5 was more expressed in the cytoplasm (Fig. 4b).

High-salt also induces the expression of NFAT5 mRNA and protein in HUVECs in a dose-dependent manner (Fig. 4c-d). In addition, the NFAT5 target gene vWF was significantly increased in high-salt-treated group compared to the control group (Additional file 1: Figure S3). Together, these results suggest that high-salt upregulates the expression of NFAT5 in ECs and promotes NFAT5 translocation from cytoplasm to nuclear in vivo *and* in vitro.

### High-salt activates NRLP3 inflammasome in ECs via NFAT5

**Fig. 5:**
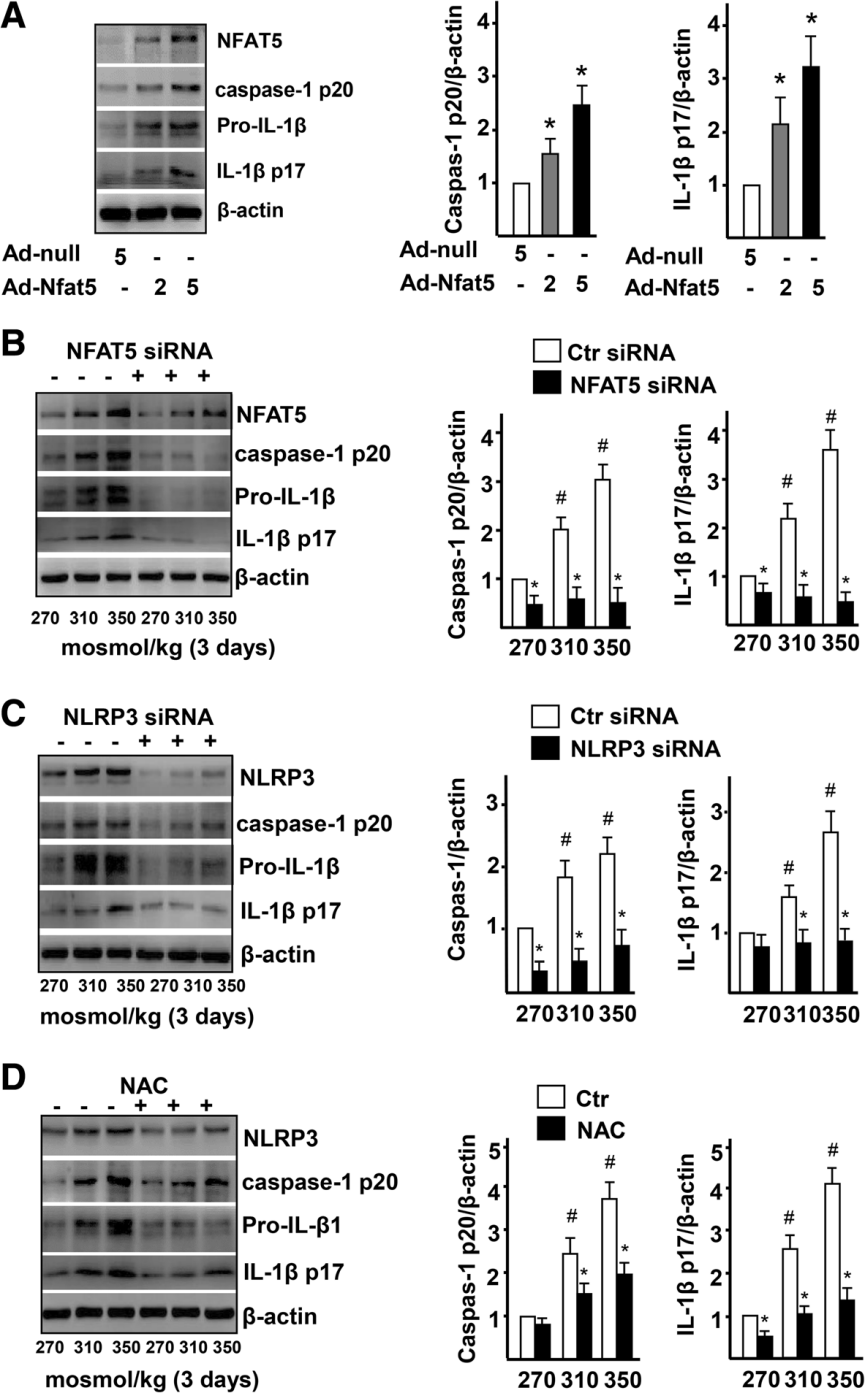
High-salt activates NRLP3 inflammasome in ECs via NFAT5. **a** Immunoblot of NFAT5, caspase-1 p20, pro-IL-1β, and IL-1β p17, and quantification of caspase-1 activity and mature IL-1β in ECs treated with Adenovirus-null (Ad-null, 5 MOI) and Adenovirus-NFAT5 (Ad-NFAT5, 2 MOI or 5 MOI). See Additional file 1: Figure S4 for caspase-1 activity. **b** Immunoblot images of NFAT5, caspase-1 p20, pro-IL-1β, and IL-1β p17, and quantification of active caspase-1 and mature IL-1β in ECs treated with high-salt and transfected with Ctr siRNA or NFAT5 siRNA. **c** Immunoblot images of NLRP3, caspase-1 p20, pro-IL-1β, and IL-1β p17, and quantification of active caspase-1 and mature IL-1β in ECs treated with high-salt and transfected with Ctr siRNA or NLRP3 siRNA. **d** Immunoblot images of NLRP3, caspase-1 p20, pro-IL-1β, and IL-1β p17, and quantification of active caspase-1 and mature IL-1β in ECs treated with high-salt, treated with NAC. All data were presented as mean ± SEM, *N* ≥ 3. *p < 0.05

To explore whether high-salt-induced NLRP3 inflammasome activation in ECs is regulated by NFAT5, we overexpressed NFAT5 by adenovirus-mediated overexpression of NFAT5 (Ad-NFAT5) in HUVECs. Western blot and statistical analysis showed that the expression of active Caspase-1 and mature IL-1β was significantly increased in the Ad-NFAT5-treated group compared to that of the control group (Ad-null, Fig. 5a). Overexpression of NFAT5 in ECs significantly increased Caspase-1 activity (Additional file 1: Figure S4), which plays a key role in the NLRP3 inflammasome-mediated cleavage of pro-IL-1β. On the contrary, when NFAT5 expression was suppressed by siRNA, the expression of active Caspase-1 and cleaved IL-1β was significantly decreased in ECs treated with high-salt (Fig. 5b). These results suggest that NFAT5 is involved in high-salt-induced NLRP3 inflammasome activation in ECs. To further examine whether NLRP3 inflammasome is a master mediator that cleaves IL-1β, we knocked down NLRP3 expression in ECs cultured with high-salt media. Western blot and statistical analysis showed that the expression of active Caspase-1 and cleaved IL-1β was significantly decreased in the NLRP3 siRNA-treated group, compared to the control group (Fig. 5c).

### High-salt-induced NFAT5 activates NRLP3 inflammasome in ECs partly via ROS

Previous study demonstrated that ROS are meditators of NLRP3 inflammasome activation [12, 24, 25]. We next evaluated the role of mitochondrial ROS in NFAT5-mediated NLRP3 inflammasome activation in HUVECs induced by high-salt. Quantification of DCFH-DA fluorescence showed that high-salt significantly increased mitochondrial ROS production in HUVECs in a dose-dependent manner (Additional file 1: Figure S5A). On the contrary, when NFAT5 expression was knocked down by siRNA, the ROS level was reduced in HUVECs treated with high-salt (Additional file 1: Figure S5B), suggesting that ROS production is mediated by NFAT5. To test whether ROS are required for high-salt-induced NLRP3 inflammasome activation in ECs, HUVECs cultured with high-salt media were treated with a ROS inhibitor NAC. The expression of active Caspase-1 and cleaved IL-1β was significantly decreased in HUVECs treated by NAC compared to the control group (Fig. 5d), indicating that the high-salt-induced NLRP3 inflammasome activation is significantly decreased when ROS production is inhibited. These results demonstrate that NFAT5 is responsible for NLRP3 inflammasome activation in ECs treated with high-salt, and ROS mediate NFAT5-induced NLRP3 inflammasome activation.

### NFAT5 directly regulates NLRP3 and IL-1β transcription in ECs

**Fig. 6:**
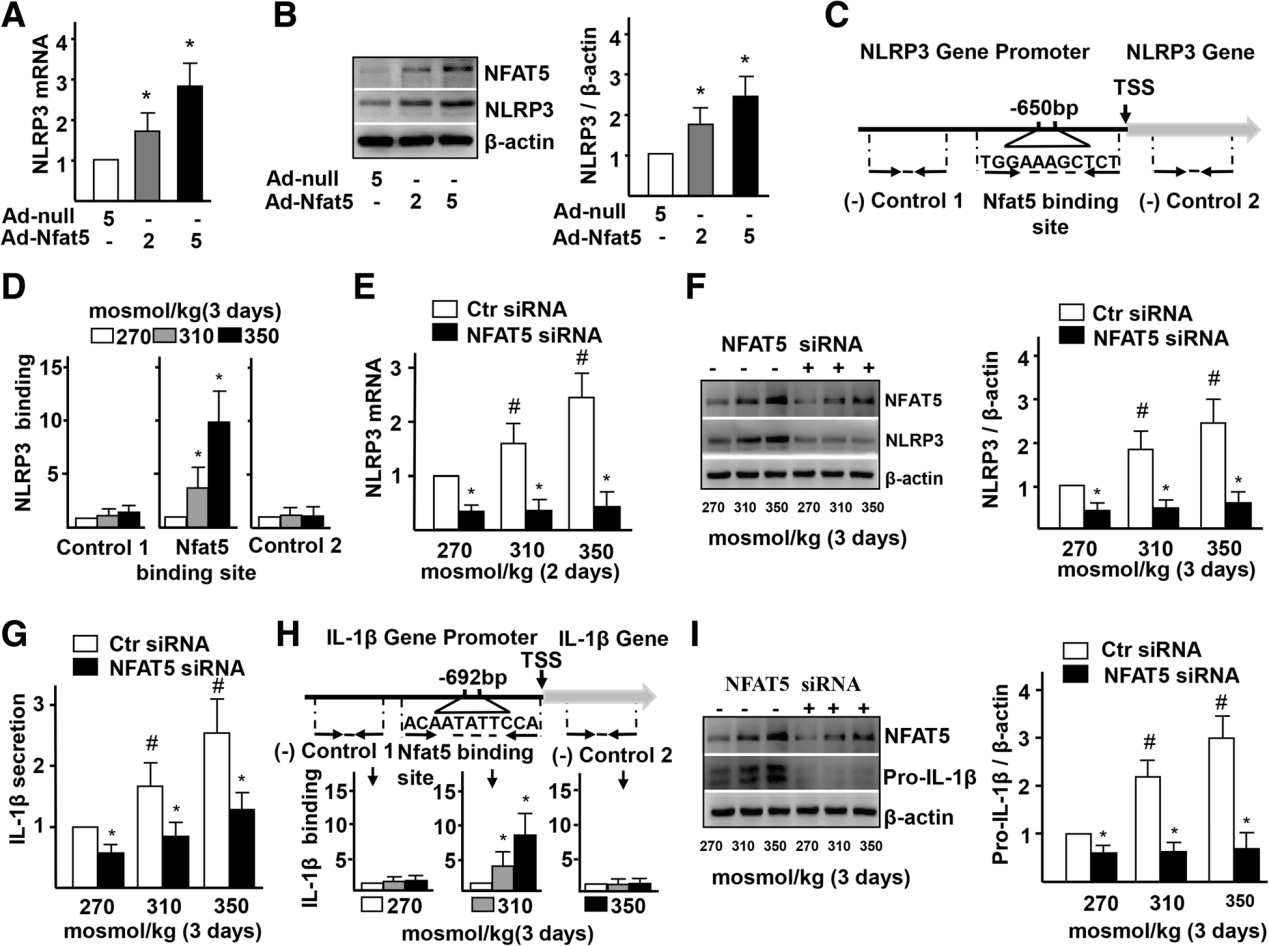
High-salt-elevated NFAT5 mediates transcription of NLRP3 and IL-1β in ECs. **a-b** mRNA and protein levels of NLRP3 in ECs treated with Adenovirus-null (Ad-null, 5 MOI) and Adenovirus-NFAT5 (Ad-NFAT5, 2 MOI or 5 MOI). **c-d** High-salt increases binding of NFAT5 to the promoter region of IL-1β. Diagram showing the region of the NFAT5 binding site upstream of the transcription start site (TSS) of NLRP3, and the regions that were used to analyze NFAT5 binding by ChIP. ChIP results are relative to 270 mosmol/kg. **e-f** mRNA and protein levels of NLRP3 in ECs treated with high-salt and transfected with Ctr siRNA or NFAT5 siRNA. **g** Protein secretion of IL-1β in ECs treated with high-salt and transfected with Ctr siRNA or NFAT5 siRNA. **h** High-salt increases binding of NFAT5 to the promoter region of IL-1β. **i** Protein levels of pro-IL-1β in ECs treated with high-salt and transfected with Ctr siRNA or NFAT5 siRNA. All data were presented as mean ± SEM, *N* ≥ 3. *p < 0.05

We next investigated the direct relationship between NFAT5, NLRP3, and IL-1β. Real-time PCR and western blot results showed that the expression of NLRP3 mRNA and protein was significantly increased in Ad-NFAT5-infected HUVECs, compared to the control group (Fig. 6a-b). To further elucidate how NFAT5 regulates the transcription of NLRP3 mRNA in ECs treated with high-salt, we examined whether NLRP3 is a direct target gene of NFAT5. Bioinformatic analysis predicted the presence of an osmotic response element (ORE, NFAT5 binding site, TGGAAAGCTCT) located at the transcription starting site of the NLRP3 gene (Fig. 6c). ChIP assay confirmed the increased binding of NFAT5 to the ORE of NLRP3 promoter in HUVECs cultured with high-salt media (Fig. 6d). To examine whether NFAT5 is required for high-salt-induced NLRP3 expression, we knocked down NFAT5 by siRNA in HUVECs. Real-time PCR and western blot results showed that the expression of NLRP3 mRNA and protein was significantly decreased in the NFAT5-siRNA-treated group, compared to the control group (Fig. 6e-f). In addition, the secretion of NLRP3 inflammasome-mediated IL-β responding to high-salt induction was also decreased in ECs that were treated by NFAT5-siRNA, compared to the control (Fig. 6g). These results confirmed that NFAT5 directly regulates the transcription of NLRP3 mRNA by binding to the promoter region of NLRP3, and NFAT5 is required for NLRP3 inflammasome activation in ECs under high-salt stimulation.

In addition, real-time PCR and western blot results showed that the expression of IL-1β mRNA and protein was significantly increased in Ad-NFAT5-infected HUVECs, compared to the control group (Additional file 1: Figure S6A-B). The consistent increase in expression of NFAT5 and IL-1β mRNA to the ECs and other cells prompted us to investigate whether IL-1β is a direct downstream target of NFAT5. Bioinformatic analysis also identified an ORE (NFAT5 binding site, ACAATATTCCA) located at the transcription starting site of the IL-1β gene (Fig. 6h). The increased binding of NFAT5 to the ORE of IL-1β promoter in HUVECs cultured with high-salt media was demonstrated by ChIP assay (Fig. 6h). On the contrary, the expression of IL-1β mRNA and protein was significantly decreased in HUVECs when NFAT5 expression was suppressed by siRNA (Additional file 1: Figure S6C and I). Together, these results demonstrate that direct binding of NFAT5 to the IL-1β promoter is required for the transcription of IL-1β mRNA in HUVECs.

## Discussion

**Fig. 7:**
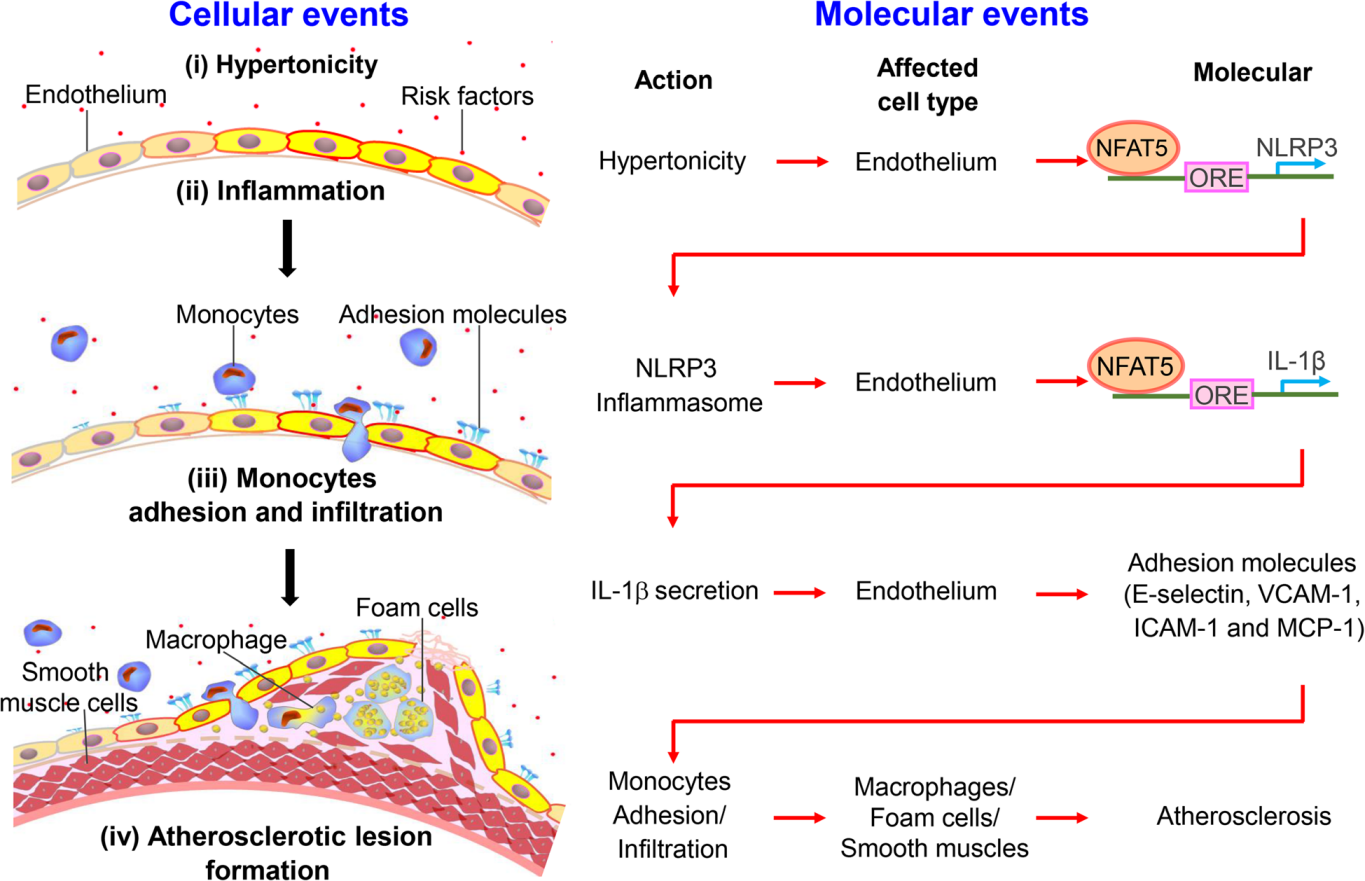
Schematic summarizes the mechanism that NLRP3 inflammasome activation in endothelium mediates hypertonic stress-induced atherosclerosis via NFAT5. Schematic illustration of the process. Stage i: Hypertonicity → NFAT5-dependent NLRP3 gene transcription → NLRP3 inflammasome activation. Stage ii: NLRP3 inflammasome activation → NFAT5-transcription-mediated pro-IL-1β → IL-1β secretion. Stage iii: IL-1β secretion → adhesive molecules → monocytes adhesion and infiltration. Stage iv: The activation of endothelial innate immunity promotes macrophage-driven foam cells and phenotype conversion of smooth muscle cells, contributing to the formation of atherosclerosis

Previous studies have proposed that the elevated concentration of serum sodium can activate vascular inflammation and contribute to AS susceptibility. A key step during the development of AS is the inflammasome activation and inflammation in ECs. However, the cellular and molecular mechanisms by which high-salt induces the inflammasome activation and inflammation in ECs remain elusive. Here, we identified that high-salt induces the expression and translocation of the transcription factor NFAT5, which is required for activating NLRP3 inflammasome-mediated inflammatory signaling pathway in ECs, leading to the accelerated macrophage infiltration and eventually AS formation. Particularly, we demonstrated that NFAT5 regulates NLRP3 expression by directly binding to its promoter region, to mediate endothelial inflammation and innate immune response under high-salt condition (Fig. 7).

Combining previous studies with the current study, it is reasonable to propose that high-salt induces AS through NFAT5 – NLRP3/NLRP3 inflammasome – IL-1β - adhesion molecules – innate immunity – AS. This process includes four major stages (Fig. 7). Stage 1, high-salt induces NFAT5-dependent NLRP3 gene transcription and NLRP3 inflammasome activation. Stage 2, NFAT5-transcription-mediated pro-IL-1β and IL-1β secretion. Stage 3, secretion of IL-1β leads to increased expression of adhesive molecules, monocytes adhesion and infiltration. Stage 4, activation of endothelial innate immunity promotes macrophage-driven foam cells and phenotype conversion of smooth muscle cells, contributing to the formation of atherosclerosis. Our study particularly highlights that in stages 1 and 2 which are the initial stages of AS, NFAT5 acts as the master regulator that is required for NLRP3 expression and NLRP3-inflammasome-mediated IL-β secretion under high-salt condition.

Our data showed that there is an increased expression and nuclear translocation of endothelial NFAT5 in AA of ApoE^−/−^ mice with high-salt intake. An important question is how high-salt intake induces endothelial NFAT5 activation. Previous studies and our results demonstrated that high-salt directly induces NFAT5 expression and nuclear translocation in endothelium in vivo and in vitro [10]. Biomechanical factors (e.g.*,* arterial wall stress), Angiotensin II as well as proinflammatory cytokines, also regulate NFAT5 expression and nuclear translocation [12, 18, 26]. In addition, many positive and negative regulators (e.g., p38, PAK, and Fyn [27–29]) have been identified in regulating NFAT5 activity. Thus, high-salt may chronically increase the blood pressure, leading to enlarged atheroprone areas by oscillatory flow shear stress and elevated arterial wall stress which synergize with those regulators to modulate NFAT5 expression and nuclear translocation.

It is reported that high-salt-induced NFAT5 promotes the inflammatory responses via p38/cFos/AP1 pathway in macrophages [30]. Interestingly, our study identified that NFAT5 translocates into the nucleus and upregulates the transcription of NLRP3 and IL-1β by binding to their promoters under the high-salt condition, leading to the activated NLRP3 inflammasome and augmented inflammation in ECs. Our results demonstrated that NFAT5 expression and translocation are required for NLRP3 and IL-1β expression in ECs subjected to high-salt treatment. Expression of NLRP3 and IL-1β is key to the composition of inflammasome and inflammation, respectively. NLRP3 inflammasome is a major mediator of innate immune responses and inflammatory cardiovascular diseases. Activation of NLRP3 inflammasome initiates ECs inflammation, and enhances macrophages infiltration and AS susceptibility [31–34]. Previous study showed that high-salt activates NLRP3 inflammasome and upregulates the expression of proinflammatory cytokines in macrophages [10]. Here, our results demonstrated that high-salt also upregulates the expression of NLRP3 mRNA and protein, and activates NLRP3 inflammasome and inflammation, enhances monocytes adhesion in ECs. These results strengthen the concept that high-salt-induced activation of endothelial NLRP3 inflammasome can initiate vascular inflammation, accelerate macrophages infiltration and AS lesion formation. Importantly, this indicates that the ECs establish a molecular threshold for the generation of AS at the transcription level.

Several molecules are involved in the assembly and activation of the NLRP3 inflammasome in ECs, including P2X7 receptor [1, 33, 35], pregnane X receptor (PXR) [36], and ROS [25, 37]. NLRP3 inflammasome detects and translates various stress stimuli into inflammatory responses. For example, some stimuli such as shear stress [21], crystals [22], and ATP [34], activate NLRP3 inflammasome in ECs and macrophages cells to mediate inflammation. Previous study also indicated that NLRP3 inflammasome is a sensor of osmotic stress in macrophages [23]. Through adaptor apoptosis-associated speck-like protein containing a CARD (ASC), NLRP3 recruits pro-caspase-1 to form inflammasome, which mediates the secretion of mature IL-1β family proteins [25, 31, 33, 38].

In the stages of AS initiation, cofactors are necessary for the activation of NLRP3 inflammasome in ECs and inflammatory AS [22, 34]. High-salt elevates ROS generation in macrophages and HEK293 cells [10, 39]. Elevated ROS production drives NLRP3 inflammasome activation in THP1 cells and macrophages [25, 32]. Our study showed that ROS generation is elevated in ECs under high-salt treatment, and the elevated ROS generation is inhibited by NFAT5 siRNA. High-salt-induced NLRP3 inflammasome activation is inhibited by a ROS inhibitor NAC. Previous study also showed that high-salt impairs NO generation and enhances Ca^2+^ influx in ECs. Impaired-nitric oxide production and enhanced Ca^2+^ influx are contributed to activating NLRP3 inflammasome [37, 40, 41]. This suggests that the generation of cofactors assists AS formation. On the other hand, these enable the tissues to increase their ability to defense harmful factors. Though how ROS are generated remains unclear in ECs under high-salt induction, recent studies demonstrated that some enzymes involved in cholesterol metabolism and consecutive oxidation reaction, regulate ROS generation and contribute to inflammatory AS [42]. In this study, we also observed that there is a significant increase of serum cholesterol in mice with high-salt intake for 12 weeks, suggesting the key enzymes of lipid metabolism probably influence high-salt-induced ROS accumulation and NLRP3 inflammasome activation in ECs. Previous studies demonstrated that cholesterol crystal induces endothelial NLRP3 inflammasome activation and promotes AS formation [22]. Our result indicates that the increase of serum cholesterol in ApoE^−/−^ mice at the late stage of high-salt intake may enhance endothelial NFAT5-mediated NLRP3 inflammasome activation. In addition, mitochondrial ROS contribute to high-salt-induced NFAT5 activation [39]. Our results showed that NFAT5-mediated ROS generation activates NLRP3 inflammasome in ECs by high-salt, indicating there may be a positive feedback loop between NFAT5 and ROS generation.

The different molecular mechanism by which NFAT5 activates inflammatory responses in different cell types during AS is reminiscent of a common concept that tissues are evolved with appropriate self-protection ability to resist risk factors that cause pathological changes. Subjected to many risk factors such as hypertonic stress, ECs undergo chronic pathological changes to form AS when the threshold is broken. In addition, it seems that the complex cellular and molecular pathways enhance the ability of blood vessels to resist the risk factors in inducing AS.

However, cardiovascular diseases account for over 30% of all global deaths, indicating the susceptibility of blood vessels to the risk factors can be out of the evolutionarily conserved self-protection ability. The prevalent of high-salt consumption worldwide, inadequate water intake, diabetes, as well as hyperosmotic therapy, all contribute to high plasma sodium concentration [2–4]. It was reported that increasing salt intake from 600 mg to 10 g per day upregulates plasma sodium by about 3 mmol/L and induces vascular disease [43]. In clinics, hypertonic saline used by hyperosmotic therapy in intracerebral hemorrhage, ischemic stroke, and hemodialysis, usually elevates the level of serum sodium up to 155 mmol/L and even higher, and causes vascular complications [44, 45]. The present study also showed that the plasma sodium concentration is increased up to 155 mmol/L in ApoE^−/−^ mice upon high-salt intake for 12 weeks, which apparently exceeds the threshold of the cells to form atherosclerotic lesions. Thus, the present study further verified the common concept at the molecular level, that reducing the risk factors such as high plasma sodium is necessary to resist cardiovascular diseases.

## Conclusions

Our findings demonstrated that hypertonic stress activates NLRP3 inflammasome-mediated innate immunity via NFAT5 in endothelium, and accelerates macrophages infiltration and AS formation. Particularly, high-salt-induced NFAT5 regulates the transcription of NLRP3 and IL-1β by binding to their promoters, and initiates NLRP3 inflammasome activation and endothelial inflammation. The findings identified in this study provide a novel mechanism of endothelial inflammation and atherosclerotic lesions formation, and suggest that ECs are the direct target cells of risk factors that initiate AS formation. The new insights into the pathogenesis of AS have clear clinical applications. As the prevalence of cardiovascular diseases all over the world, maintaining the tissues below an endurable evolutionarily endowed threshold that leads to pathological changes would go far towards keeping healthy.

## Additional file

Additional file 1: Supplementary Materials and Methods. **Figure S1.** Effect of high salt intake on weight and atherosclerotic lesions formation of ApoE-/- mice. **Figure S2.** HUVECs adapt well in hypertonic medium. Figure S3 High-salt elevates the expression of vWF in HUVECs. **Figure S4.** Overexpression of NFAT5 increases Caspase-1 activity in ECs by treated with Ad-null, 5 MOI, Ad-NFAT5, 2.5 MOI or 5 MOI. **Figure S5.** High Salt-elevated NFAT5 mediate ROS production in ECs via. **Figure S6.** NFAT5 mediates IL-1β expression. **Table S1.** The primers sequences for RT-qPCR in this study. **Table S2.** Effect of high-salt intake on serum level of ApoE-/- mice. (DOCX 1785 kb)

## Data Availability

All data generated or analyzed during this study are included in this published article and its supplementary information files.

## Abbreviations

AA: Aortic arch
ApoE^−/−^ mice: Apolipoprotein E-deficient mice
ASC: Apoptosis-associated speck-like protein containing a CARD
ChIP: Chromatin immunoprecipitation
ECs: Endothelial cells
HUVECs: Human umbilical vein endothelial cells
ICAM-1: Intercellularcelladhesionmolecule-1
IL-18: Interleukin-18
IL-1β: Interleukin -1β
LDL: Low density lipoprotein
MCP-1: Monocyte chemoattractant protein-1
NAC: N-acetylcysteine
NFAT5: Nuclear factor of activated T cells 5
NLRP3: NLR family pyrin domain containing 3
ORE: Osmotic response element
TA: Thoracic aorta
VCAM-1: Vascular cell adhesion molecule-1
β-ECGF: β-Endothelial cell growth factor
