# Supplementary material for: NFAT5 mediates hypertonic stress-induced atherosclerosis via activating NLRP3 inflammasome in endothelium
Source: Cell Commun Signal. 2019 Aug 20;17:102. doi: 10.1186/s12964-019-0406-7 (PMC6701070; doi:10.1186/s12964-019-0406-7)
Supplement: Supplementary file 1 — Supplementary Materials and Methods. Figure S1. Effect of high salt intake on weight and atherosclerotic lesions formation of ApoE-/- mice. Figure S2. HUVECs adapt well in hypertonic medium. Figure S3 High-salt elevates the expression of vWF in HUVECs. Figure S4. Overexpression of NFAT5 increases Caspase-1 activity in ECs by treated with Ad-null, 5 MOI, Ad-NFAT5, 2.5 MOI or 5 MOI. Figure S5. High Salt-elevated NFAT5 mediate ROS production in ECs via. Figure S6. NFAT5 mediates IL-1β expression. Table S1. The primers sequences for RT-qPCR in this study. Table S2. Effect of high-salt intake on serum level of ApoE-/- mice. (DOCX 1785 kb) [file 12964_2019_406_MOESM1_ESM.docx]

**Supplementary information**

**NFAT5** **mediates hypertonic stress-induced atherosclerosis via activating NLRP3 inflammasome in endothelium**

Pingping Ma^a^, Shenfang Zha^a^, Xinkun Shen^a^, Yulan Zhao^a^, Li Li^a^, Li Yang^a^, Mingxing Lei^b,c,*^ & Wanqian Liu^a,*^

^a^ Key Laboratory of Biorheological Science and Technology, Ministry of Education, Bioengineering College, Chongqing University, Chongqing 400044, China.

^b^ Integrative Stem Cell Center, China Medical University Hospital, China Medical University, Taichung 40402, Taiwan.

^c^ Institute of New Drug Development, College of Biopharmaceutical and Food Sciences, China Medical University, Taichung 40402, Taiwan.

Correspondence should be addressed to W.L. (wqliu@cqu.edu.cn) or M.L. (mingxing@mail.cmu.edu.tw)

The supplementary information file contains Materials and Methods, supplementary tables (Table S1 to S2) and supplementary figures (supplementary figures 1 to 6).

**Materials and Methods**

**NAC Treatment**

HUVECs were cultured in 6-well plates overnight, then treated by 5 mM N-Acetyl-L-cysteine (NAC) (Sigma, Billerica, MA) for 2 h before stimulated with NaCl. After 24 h, the protein was extracted for western blot.

**Intracellular ROS quantification.**

The level of intracellular reactive oxygen species (ROS) was quantitated by the change of fluorescent probe 2′,7′-dichlorofluorescin diacetate (DCFH-DA) (Sigma, Billerica, MA). Briefly, HUVECs were planted, then treated with control or NFAT5 siRNA (50 nM) followed by NaCl, or planted HUVECs were just stimulate by different high salt medium for 2 days. Cells were washed twice with PBS, and incubated with 10 μM DCFH-DA for 30 min at 37 °C in dark. Subsequently, cells were washed by PBS for two times and analyzed by the microscope (Leica, Germany).

**Monocyte-HUVEC adhesion assay.**

HUVECs planted in 6-wells plates were treated with control or NLRP3 siRNA followed by NaCl as described above for siRNA transfection. Human THP-1 monocytes were washed with PBS twice and labeled with Calcein (Invitrogen, Carlsbad, CA) to distinguish them from HUVECs. Subsequently, added adequate complete medium to make the monocytes concentration reach 3.0×10^5^ per ml. After divided into 6-wells plates equally, monocytes were co-incubated with stimulated HUVECs at 37 °C for 30 min. Rinsed them twice with PBS before taking pictures.

**Hematoxylin-eosin staining.**

Mice were euthanized and perfused by PBS and paraformaldehyde successively. Then the aortas were fixed, paraffin embedded, and mounted in the microtome. Consecutive sections (5 µm thickness) were taken from aortic arch, and stained with hematoxylin/eosin. Pictures were obtained by the microscope.

**Enzyme linked immunosorbent assay (ELISA).**

HUVECs were exposed to high NaCl in 2% serum medium. Then, the media was collected and centrifuged at 1,000 × g for 15 min. After that, IL-1β secretion was measured by using the Human IL-1β ELISA Kit (Neo Bioscience, Shenzhen, GD) in the supernatants. In the same time, HUVECs were rinsed and lysed, and total cell protein concentrations were measured as described above for Western blot. The amount of IL-1β secreted in supernatant was calculated from the concentration of IL-1β, normalized by respective total protein. Finally, the rate of secretion of IL-1β in hyperosmotic medium was normalized by it in control medium. Caspase-1 activity was measured by using the Human Caspase-1 ELISA Kit (Abcam, Cambridge, MA) as described above, according to the manufacturer's protocol.

Blood samples were collected into new tubes from mice. Let them rest 30 min at room temperature. Afterwards, tubes were centrifuged at 1,800 × g for 15 min at 4 °C to remove the clot. The serum was transferred into new tubes, and serum levels of IL-1β were detected by using the Mouse IL-1β ELISA Kit (Neo Bioscience, Shenzhen, GD), as per standard procedures.

**Supplementary figures and legends**

**
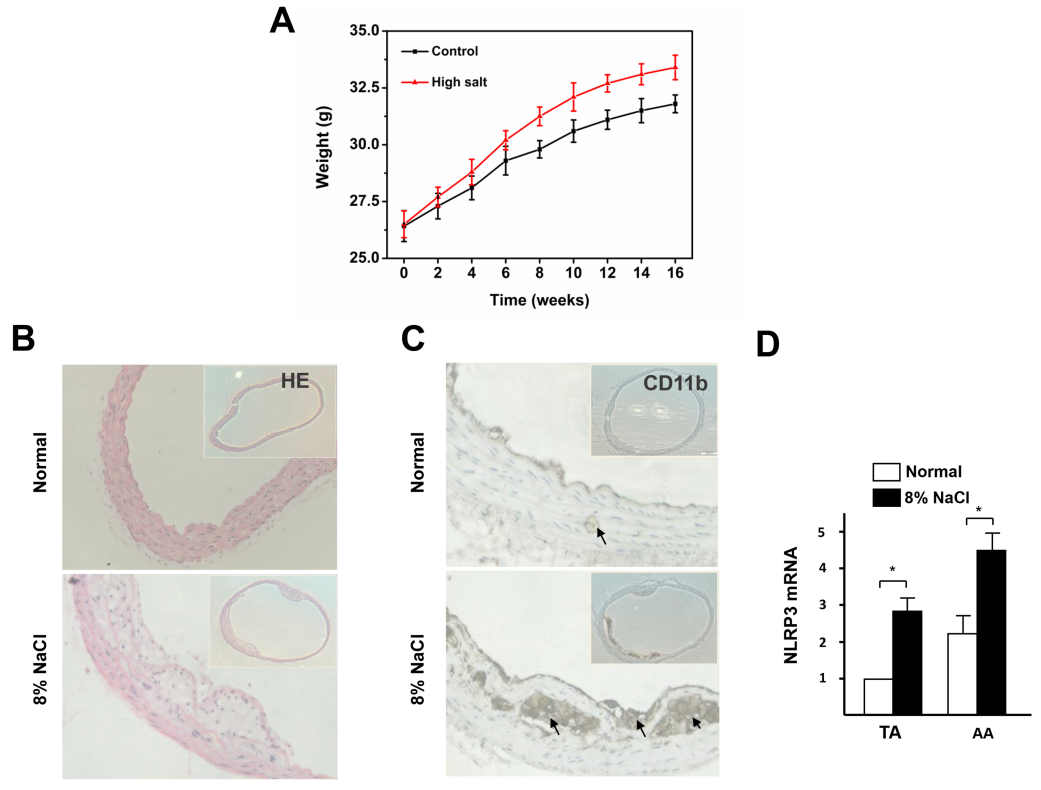
**

**Figure S1** Effect of high salt intake on weight and atherosclerotic lesions formation of ApoE^-/-^ mice. (**A**) Quantification of weight of ApoE^-/-^ mice fed with normal or high-salt diet. (**B**) Representative hematoxylin-eosin staining of cross-sections of AA from ApoE^-/-^ mice. (**C**) Representative immunohistochemistry of cross-sections of AA from ApoE^-/-^ mice with an antibody against the macrophage marker CD-11b (n = 9). Arrows indicate the CD-11b-positive macrophages. (**D**) Quantification of mRNA levels of NLRP3 in TA and AA of ApoE^-/-^ mice fed with normal or high-salt diet. All data were presented as mean ± SEM, N≥3. *p < 0.05.


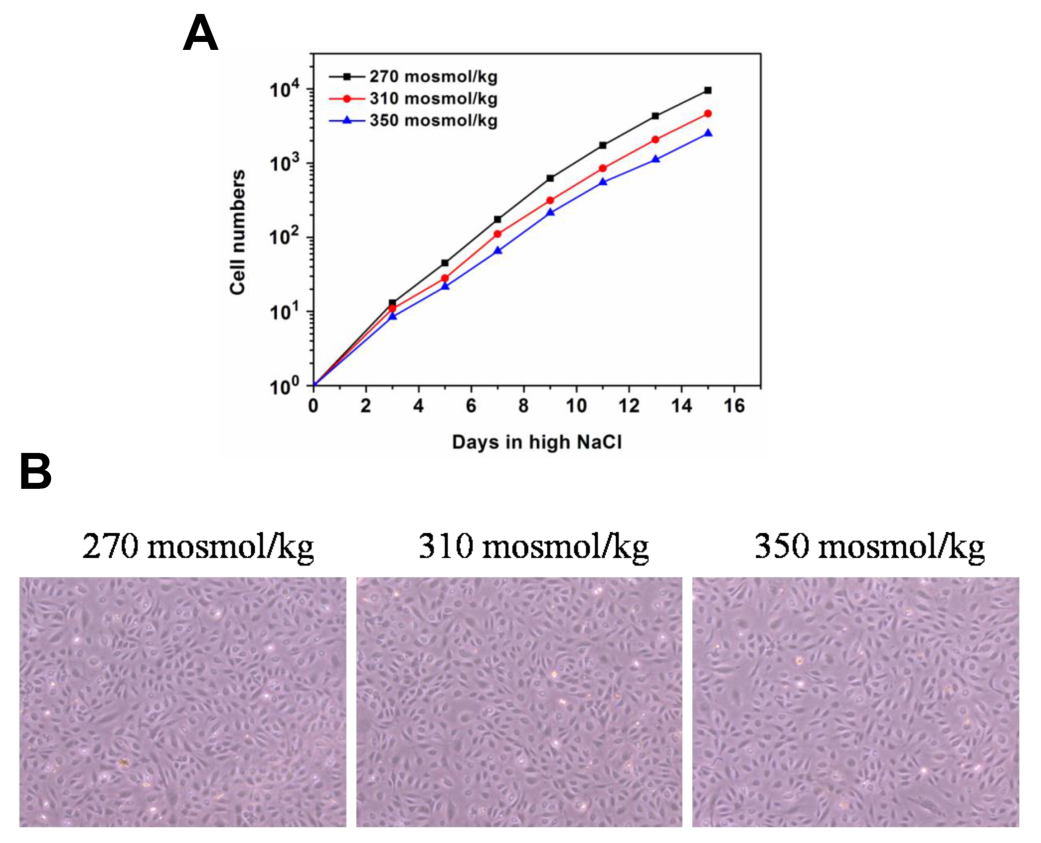


**Figure S2** HUVECs adapt well in hypertonic medium. (**A**) ECs maintain logarithmic growth in hypertonic medium. (**B**) Images of ECs cultured in iso- and hyper-osmotic medium for 15 d.


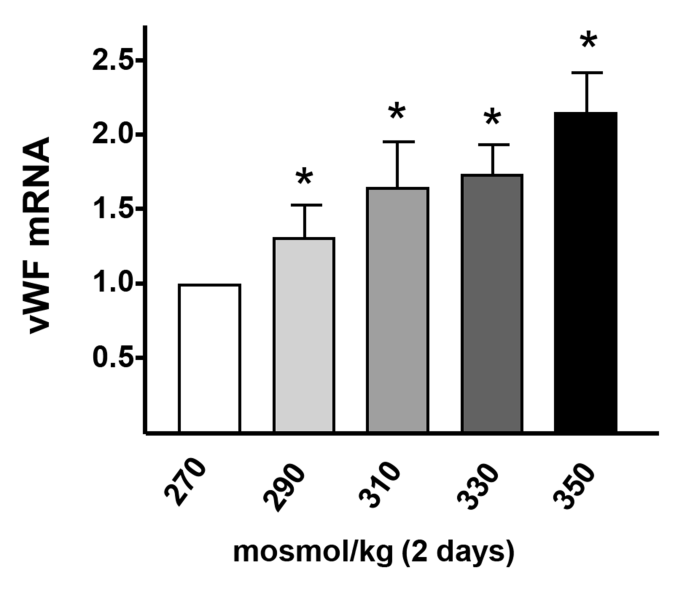


**Figure S3** High-salt elevates the expression of vWF in HUVECs. All data were presented as mean ± SEM, N≥3. *p < 0.05..


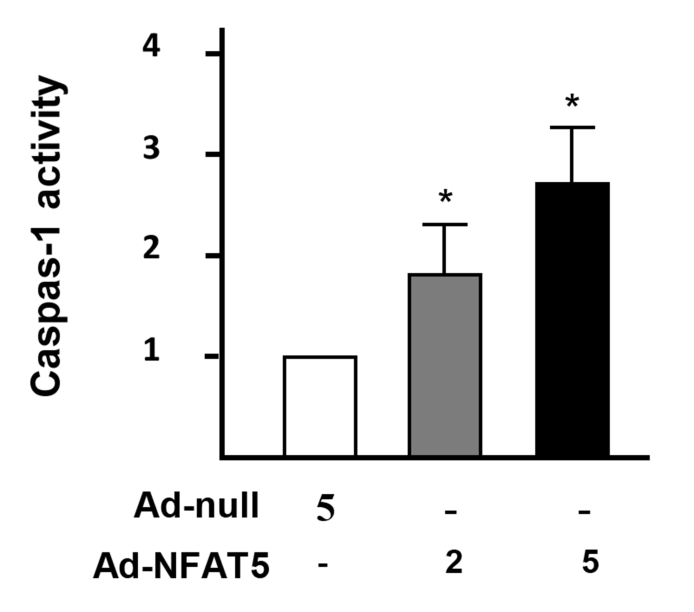


**Figure S4** Overexpression of NFAT5 increases Caspase-1 activity in ECs by treated with Ad-null, 5 MOI, Ad-NFAT5, 2.5 MOI or 5 MOI. All data were presented as mean ± SEM, N≥3. *p < 0.05.


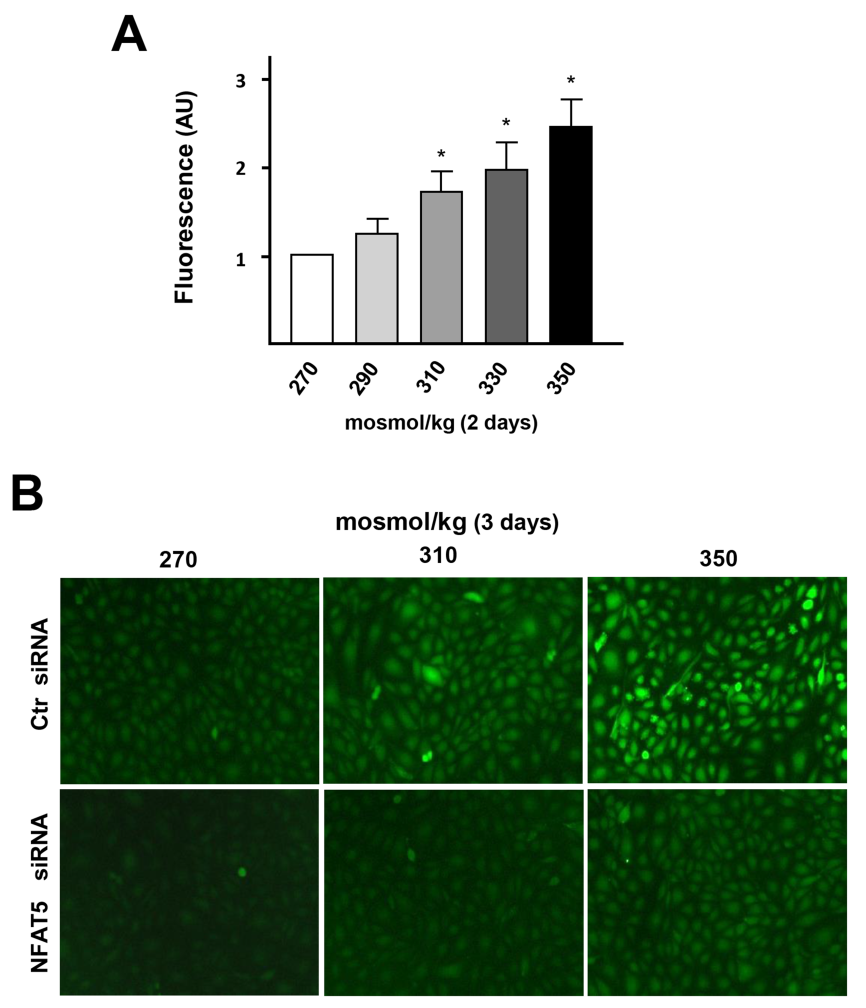


**Figure S5** High Salt-elevated NFAT5 mediate ROS production in ECs via. (**A**) ROS production monitored by measuring DCFH-DA fluorescence in HUVECs stimuli by different hyper-osmotic medium. (**B**) ROS fluorescent activity in HUVECs at hyper-osmotic medium transfected with Ctr siRNA or NFAT5 siRNA, compared with isomolar controls. All data were presented as mean ± SEM, N≥3. *p < 0.05.


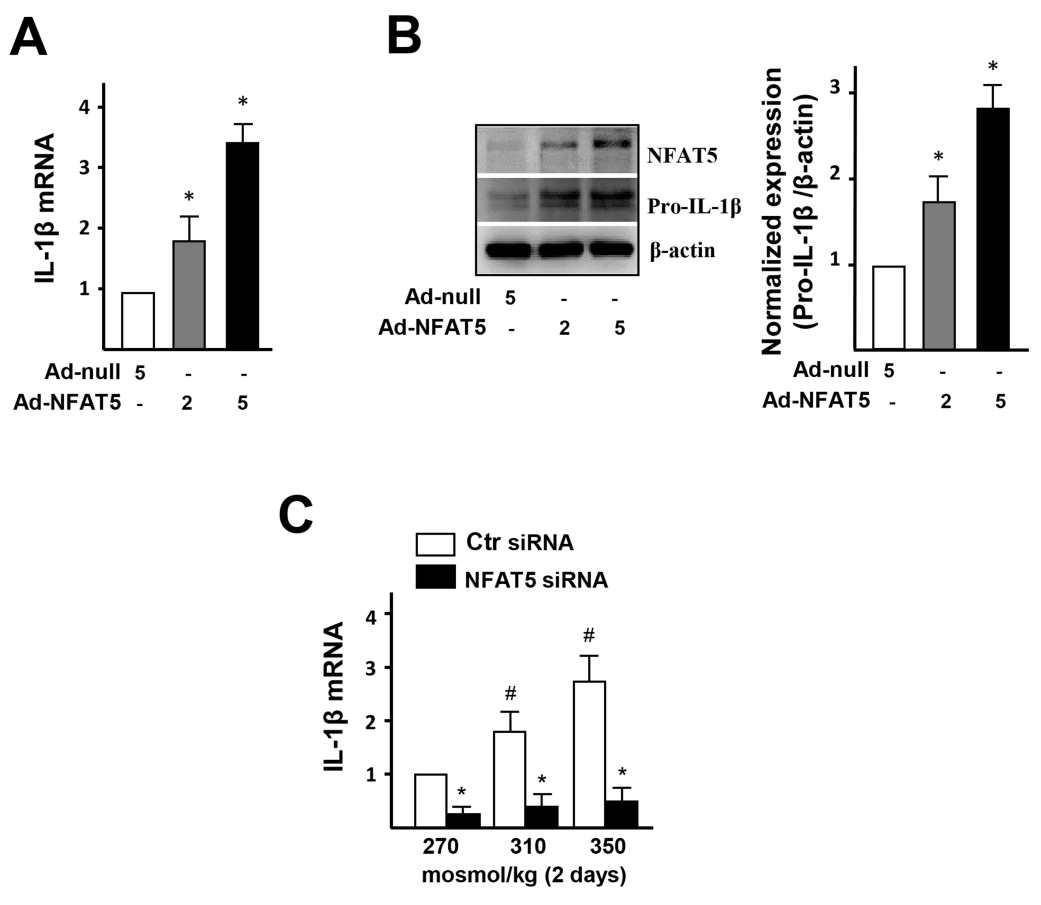


**Figure S6** NFAT5 mediates IL-1β expression. (**A-B**) mRNA and protein levels of IL-1β in ECs treated with Adenovirus-null (Ad-null, 5 MOI) and Adenovirus-NFAT5 (Ad-NFAT5, 2 MOI or 5 MOI). (**C**) mRNA levels of IL-1β in ECs by high NaCl, transfected with Ctr siRNA or NFAT5 siRNA. All data were presented as mean ± SEM, N≥3. *p < 0.05.

**Supplementary Tables.**

**Table S1**. The primers sequences for RT-qPCR in this study.

| ***Target genes*** | ***Primer sequences*** |
| --- | --- |
| Human NFAT5  Human NLRP3  Human IL-1β  Human β-actin  Human vWF | **Forward:** 5’- ACAGTAAAGCTGGAAGGCCA -3’  **Reverse:** 5’- TTGCTAGGATCAAGGCCGAC -3’  **Forward:** 5’ **–** CTTCTCTGATGAGGCCCAAG -3 ’  **Reverse:** 5’ **–** GCAGCAAACTGGAAAGGAAG -3’  **Forward:** 5’- CTCTGGGATTCTCTTCAGCCA -3 ’  **Reverse:** 5’ – AGGAGCACTTCATCTGTTTAGGG -3 ’  **Forward:** 5’- CGAGCGCGGCTACAGCTT -3’  **Reverse:** 5’ - TCCTTAATGTCACGCACGATTT -3’  **Forward:** 5’ - CCTTGACCTCGGACCCTTATG -3 ’  **Reverse:** 5’ - GATGCCCGTTCACACCACT -3’ |
| Human ICAM-1 | **Forward:** 5’- ATGCCCAGACATCTGTGTCC -3’  **Reverse:** 5’- GGGGTCTCTATGCCCAACAA -3’ |
| Human VCAM-1 | **Forward:** 5’- GGGAAGATGGTCGTGATCCTT -3’  **Reverse:** 5’- TCTGGGGTGGTCTCGATTTTA -3’ |
| Human E-sele  Human MCP-1 | **Forward:** 5’- AGAGTGGAGCCTGGTCTTACA -3’  **Reverse:** 5’- CCTTTGCTGACAATAAGCACTGG -3’  **Forward:** 5’- AGCATGAAAGTCTCTGCCGCCCTTCTG -3’  **Reverse:** 5’- ATTACTTAAGGCATAATGTTTCACA -3 |
| Mouse NFAT5  Mouse NLRP3  Mouse IL-1β  Mouse GAPDH    Mouse ICAM-1  Mouse VCAM-1  Mouse E-selectin  Mouse MCP-1 | **Forward:** 5’- ATCGCCCAAGTCCCTGTACT -3’  **Reverse:** 5’- GCTTGTCTGACTCATTGATGCTA -3’  **Forward:** 5’ **-** ATTACCCGCCCGAGAAAGG -3 ’  **Reverse:** 5’ **-** TCGCAGCAAAGATCCACACAG -3’  **Forward:** 5’- GCAACTGTTCCTGAACTCAACT -3 ’  **Reverse:** 5’ - ATCTTTTGGGGTCCGTCAACT -3 ’  **Forward:** 5’- AGGTCGGTGTGAACGGATTTG -3’  **Reverse:** 5’ - GGGGTCGTTGATGGCAACA -3’  **Forward:** 5’- GTGATGCTCAGGTATCCATCCA -3’  **Reverse:** 5’- CACAGTTCTCAAAGCACAGCG -3’  **Forward:** 5’- TTGGGAGCCTCAACGGTACT -3’  **Reverse:** 5’- GCAATCGTTTTGTATTCAGGGGA -3’  **Forward:** 5’- CCAATCTGAAACATTCACCGAGT -3’  **Reverse:** 5’- GAGTCTTTGGTTCGTTGGATGTA -3’  **Forward:** 5’- TTAAAAACCTGGATCGGAACCAA -3’  **Reverse:** 5’- GCATTAGCTTCAGATTTACGGGT -3’ |

**Table S2**. Effect of high-salt intake on serum level of ApoE^-/-^ mice.

| **Serum variable**   **Control** **8% NaCl** |
| --- |
| Sodium, mmol/L 150 ± 0.9 155 ± 1.4^*^  Potassium, mmol/L 8.94 ± 0.2 8.37 ± 0.1^*^  Glucose, mmol/L 5.9 ± 0.3 6.4 ± 0.4  Urea, mmol/L 10.3 ± 1.7 13.7 ± 1.1^*^  Cholesterol, mmol/L 4.45 ± 0.8 7.45 ± 1.2^*^ |
